# Knowledge, attitudes and clinical practice regarding behavioral treatments and psychological issues in migraine: a survey of AHS members

**DOI:** 10.1186/1129-2377-14-S1-P61

**Published:** 2013-02-21

**Authors:** DC Buse, F Andrasik, RB Lipton, CM Sollars, AD McMichael, RA Nicholson

**Affiliations:** 1 Albert Einstein College of Medicine, USA; 2 University of Memphis, USA; 3 Montefiore Headache Center, USA; 4 Mercy Clinic Headache Center, USA

## Background and objectives

We aimed to gather data regarding knowledge, attitudes, and clinical practices related to behavioral treatments for migraine and psychological issues among healthcare professionals (HCPs), an area about which little is currently known.

## Methods

784 American Headache Society (AHS) members were asked to complete a web-based survey. Data on sociodemographics, clinical practice patterns, attitudes, and knowledge were collected. Analyses contrasted HCPs’ (physicians [MD], psychologists/mental health professionals [PSY], and nurse practitioners/physician’s assistants [NP/PA]) knowledge of empirical evidence for efficacy of behavioral treatments, assessment/referral practices, and related beliefs.

## Results

The 134 respondents were comprised of MDs (74%), PSYs(12%), and NP/PAs(14%). Knowledge that certain behavioral treatments have “Grade A” evidence for migraine prevention was highest among PSYs for biofeedback (p<0.01), cognitive behavioral therapy ( p<.001), and relaxation training (p<.001). The majority of respondents reported that they routinely assess headache patients for depression (82.9%) and anxiety (69.2%). 30.8% routinely assessed abuse/PTSD. The overall referral rate for non-pharmacologic treatment was below 20%, with stress management, relaxation training, and psychotherapy being the most common referrals. The probability of referring a patient for behavioral treatment is correlated with knowledge regarding US Headache Consortium guidelines and availability of behavioral treatment in the respondent’s geographic region.

## Conclusions

Other than psychologists, the majority of respondents were unaware that behavioral treatments possess “Grade A” evidence for migraine prevention according to US Headache Consortium guidelines. Low rates of referrals for behavioral treatments may result from a combination of knowledge and beliefs and a lack of available services. A need exists for education regarding the empirical evidence supporting the efficacy of certain behavioral treatments in migraine management.

